# Supplementary material for: Deletion of wfs1 Impairs Oligodendrocyte Precursor Cells Dorsal Distribution and Myelination Through the wfs1-hmgcs1 Axis in Zebrafish
Source: Biology (Basel). 2026 Aug 16;15(16):1402. doi: 10.3390/biology15161402 (PMC13510151; doi:10.3390/biology15161402)
Supplement: Supplementary file 1 [file biology-15-01402-s001.zip › File S1-Transcriptome sequencing data and differentially expressed genes (DEGs) in wfs1-- zebrafish at 5 dpf/2.KEGG/wfs1KOvsWT/GSEA/wfs1KO/SALMONELLA_INFECTION/SALMONELLA_INFECTION(DRE05132).html]

Details for gene set SALMONELLA\_INFECTION(DRE05132)[GSEA]

|  || Dataset | deg.sample |
| Phenotype | sample.cls |
| Upregulated in class | wfs1KO |
| GeneSet | SALMONELLA\_INFECTION(DRE05132) |
| Enrichment Score (ES) | 0.23686674 |
| Normalized Enrichment Score (NES) | 1.5002216 |
| Nominal p-value | 0.0 |
| FDR q-value | 0.21956567 |
| FWER p-Value | 0.637 |
Table: GSEA Results Summary

  

Fig 1: Enrichment plot: SALMONELLA\_INFECTION(DRE05132)      
 Profile of the Running ES Score & Positions of GeneSet Members on the Rank Ordered List

  

| PROBE | DESCRIPTION (from dataset) | GENE SYMBOL | GENE\_TITLE | RANK IN GENE LIST | RANK METRIC SCORE | RUNNING ES | CORE ENRICHMENT || 1 | 57934\_1 | actb1 |  |  | 1 | 4.982 | 0.0187 | Yes |
| 2 | 386590\_1 | hsp90b1 |  |  | 4 | 4.977 | 0.0374 | Yes |
| 3 | 563023\_1 | flnb |  |  | 34 | 4.927 | 0.0550 | Yes |
| 4 | 562529\_1 | flna |  |  | 42 | 4.912 | 0.0733 | Yes |
| 5 | 101882034 | LOC101882034 |  |  | 73 | 4.809 | 0.0905 | Yes |
| 6 | 566185 | caspbl |  |  | 88 | 4.759 | 0.1080 | Yes |
| 7 | 100002872\_2 | cyfip2 |  |  | 265 | 3.827 | 0.1168 | Yes |
| 8 | 562372\_1 | snx18a |  |  | 405 | 3.283 | 0.1247 | Yes |
| 9 | 100004870\_1 | exoc2 |  |  | 454 | 3.114 | 0.1349 | Yes |
| 10 | 548603\_1 | snx18b |  |  | 558 | 2.830 | 0.1423 | Yes |
| 11 | 570238 | fosaa |  |  | 589 | 2.782 | 0.1518 | Yes |
| 12 | 561021 | si:dkey-103e21.5 |  |  | 599 | 2.768 | 0.1620 | Yes |
| 13 | 556226\_1 | cdc42l2 |  |  | 680 | 2.623 | 0.1693 | Yes |
| 14 | 793158\_1 | cdc42l |  |  | 826 | 2.351 | 0.1736 | Yes |
| 15 | 562552 | zgc:171775 |  |  | 839 | 2.330 | 0.1820 | Yes |
| 16 | 677750 | myl2b |  |  | 901 | 2.226 | 0.1884 | Yes |
| 17 | 436718 | rhocb |  |  | 948 | 2.171 | 0.1951 | Yes |
| 18 | 558917\_1 | snx9b |  |  | 1039 | 2.055 | 0.2000 | Yes |
| 19 | 798985\_1 | vps16 |  |  | 1110 | 1.984 | 0.2052 | Yes |
| 20 | 393653\_1 | rhogd |  |  | 1332 | 1.780 | 0.2049 | Yes |
| 21 | 110437957 | LOC110437957 |  |  | 1463 | 1.684 | 0.2071 | Yes |
| 22 | 571356 | pik3c2a |  |  | 1634 | 1.580 | 0.2077 | Yes |
| 23 | 394198 | fosab |  |  | 1745 | 1.508 | 0.2099 | Yes |
| 24 | 101882911 | LOC101882911 |  |  | 1827 | 1.446 | 0.2128 | Yes |
| 25 | 431777 | tuba7l |  |  | 2071 | 1.338 | 0.2101 | Yes |
| 26 | 373080 | tuba1b |  |  | 2072 | 1.337 | 0.2151 | Yes |
| 27 | 30429\_1 | mylpfa |  |  | 2177 | 1.294 | 0.2167 | Yes |
| 28 | 566604 | casp3b |  |  | 2287 | 1.253 | 0.2179 | Yes |
| 29 | 373107 | birc2 |  |  | 2293 | 1.250 | 0.2225 | Yes |
| 30 | 565563 | kif5c |  |  | 2416 | 1.202 | 0.2231 | Yes |
| 31 | 563560 | ikbkb |  |  | 2417 | 1.202 | 0.2277 | Yes |
| 32 | 563632\_1 | wasf3b |  |  | 2486 | 1.178 | 0.2299 | Yes |
| 33 | 556873\_1 | si:ch211-175f11.5 |  |  | 2786 | 1.085 | 0.2245 | Yes |
| 34 | 571356\_2 | pik3c2a |  |  | 2879 | 1.063 | 0.2256 | Yes |
| 35 | 101884483 | LOC101884483 |  |  | 2886 | 1.061 | 0.2294 | Yes |
| 36 | 373874 | ripk2 |  |  | 3040 | 1.027 | 0.2284 | Yes |
| 37 | 100009637\_2 | wasla |  |  | 3137 | 1.004 | 0.2292 | Yes |
| 38 | 110439888 | LOC110439888 |  |  | 3177 | 0.998 | 0.2317 | Yes |
| 39 | 573216 | tuba1a |  |  | 3240 | 0.984 | 0.2334 | Yes |
| 40 | 406820 | actr2a |  |  | 3314 | 0.969 | 0.2347 | Yes |
| 41 | 449788 | s100a10a |  |  | 3362 | 0.961 | 0.2369 | Yes |
| 42 | 336637 | zgc:56493 |  |  | 3583 | 0.914 | 0.2333 | No |
| 43 | 564295\_1 | traf2a |  |  | 3833 | 0.862 | 0.2287 | No |
| 44 | 337223 | tuba8l |  |  | 3910 | 0.846 | 0.2294 | No |
| 45 | 406270 | acbd3 |  |  | 4028 | 0.823 | 0.2288 | No |
| 46 | 30429 | mylpfa |  |  | 4031 | 0.822 | 0.2318 | No |
| 47 | 407638 | dctn1a |  |  | 4111 | 0.809 | 0.2324 | No |
| 48 | 100003363 | rab9b |  |  | 4215 | 0.791 | 0.2321 | No |
| 49 | 795671\_1 | tlr4al |  |  | 4308 | 0.775 | 0.2321 | No |
| 50 | 559154 | ptprc |  |  | 4330 | 0.772 | 0.2343 | No |
| 51 | 100003859 | cxcl8b.3 |  |  | 4445 | 0.753 | 0.2335 | No |
| 52 | 403125 | tlr2 |  |  | 4573 | 0.736 | 0.2323 | No |
| 53 | 793648 | LOC793648 |  |  | 4799 | 0.705 | 0.2278 | No |
| 54 | 751630 | zgc:153713 |  |  | 4826 | 0.702 | 0.2296 | No |
| 55 | 100004321 | LOC100004321 |  |  | 5124 | 0.665 | 0.2227 | No |
| 56 | 101884351 | LOC101884351 |  |  | 5336 | 0.644 | 0.2184 | No |
| 57 | 564295 | traf2a |  |  | 5504 | 0.634 | 0.2155 | No |
| 58 | 323099 | nfkbiab |  |  | 5568 | 0.630 | 0.2159 | No |
| 59 | 450032 | zgc:101785 |  |  | 5599 | 0.626 | 0.2173 | No |
| 60 | 402901 | dynlt3 |  |  | 5666 | 0.620 | 0.2175 | No |
| 61 | 436734 | txn |  |  | 5716 | 0.615 | 0.2183 | No |
| 62 | 406486 | m6pr |  |  | 5789 | 0.608 | 0.2183 | No |
| 63 | 100002318 | mapk13 |  |  | 6155 | 0.572 | 0.2088 | No |
| 64 | 393711 | abi1a |  |  | 6315 | 0.555 | 0.2059 | No |
| 65 | 798868 | LOC798868 |  |  | 6449 | 0.542 | 0.2037 | No |
| 66 | 101884425 | LOC101884425 |  |  | 6612 | 0.527 | 0.2005 | No |
| 67 | 393452 | dync2li1 |  |  | 6621 | 0.526 | 0.2022 | No |
| 68 | 100007897 | pkn1b |  |  | 6685 | 0.521 | 0.2022 | No |
| 69 | 403148 | tirap |  |  | 6709 | 0.518 | 0.2034 | No |
| 70 | 405821 | rab5b |  |  | 6719 | 0.518 | 0.2051 | No |
| 71 | 562529 | flna |  |  | 6775 | 0.513 | 0.2053 | No |
| 72 | 100330514 | pik3c2g |  |  | 6869 | 0.503 | 0.2042 | No |
| 73 | 30686 | myca |  |  | 6877 | 0.502 | 0.2059 | No |
| 74 | 448942 | vps33a |  |  | 6966 | 0.494 | 0.2050 | No |
| 75 | 564048 | plekhm1 |  |  | 6976 | 0.493 | 0.2065 | No |
| 76 | 65236 | mapk8b |  |  | 6998 | 0.492 | 0.2077 | No |
| 77 | 403133 | tlr18 |  |  | 7008 | 0.491 | 0.2093 | No |
| 78 | 58130 | tradd |  |  | 7033 | 0.489 | 0.2104 | No |
| 79 | 57923 | pycard |  |  | 7045 | 0.488 | 0.2118 | No |
| 80 | 792024 | si:ch73-199e17.1 |  |  | 7117 | 0.481 | 0.2114 | No |
| 81 | 561737 | pik3ca |  |  | 7195 | 0.474 | 0.2107 | No |
| 82 | 386807 | rab5c |  |  | 7243 | 0.470 | 0.2110 | No |
| 83 | 393154 | tuba8l4 |  |  | 7289 | 0.467 | 0.2113 | No |
| 84 | 335916 | jun |  |  | 7293 | 0.467 | 0.2130 | No |
| 85 | 403145 | myd88 |  |  | 7314 | 0.466 | 0.2141 | No |
| 86 | 799806\_1 | anxa2b |  |  | 7447 | 0.457 | 0.2117 | No |
| 87 | 795671 | tlr4al |  |  | 7485 | 0.455 | 0.2122 | No |
| 88 | 103909610 | LOC103909610 |  |  | 7518 | 0.453 | 0.2129 | No |
| 89 | 108191324 | LOC108191324 |  |  | 7553 | 0.451 | 0.2135 | No |
| 90 | 100151249 | stx10 |  |  | 7650 | 0.442 | 0.2121 | No |
| 91 | 108191324\_1 | LOC108191324 |  |  | 7769 | 0.439 | 0.2100 | No |
| 92 | 562552\_1 | zgc:171775 |  |  | 7885 | 0.432 | 0.2080 | No |
| 93 | 100006132 | si:ch211-1f22.2 |  |  | 8076 | 0.417 | 0.2035 | No |
| 94 | 393902\_2 | rab7 |  |  | 8135 | 0.416 | 0.2033 | No |
| 95 | 553542\_1 | zgc:109889 |  |  | 8314 | 0.412 | 0.1992 | No |
| 96 | 101886613 | si:ch211-63p21.2 |  |  | 8353 | 0.411 | 0.1995 | No |
| 97 | 406598 | pik3cg |  |  | 8441 | 0.407 | 0.1983 | No |
| 98 | 336504 | arpc5a |  |  | 8597 | 0.395 | 0.1948 | No |
| 99 | 572690 | dynll2b |  |  | 8704 | 0.388 | 0.1929 | No |
| 100 | 65239 | map2k6 |  |  | 8937 | 0.372 | 0.1870 | No |
| 101 | 560913 | map2k7 |  |  | 9023 | 0.366 | 0.1856 | No |
| 102 | 406463 | nfkbiaa |  |  | 9066 | 0.363 | 0.1857 | No |
| 103 | 378972 | akt2 |  |  | 9293 | 0.347 | 0.1798 | No |
| 104 | 553788 | map3k7 |  |  | 9427 | 0.338 | 0.1768 | No |
| 105 | 555437 | si:ch73-362m14.4 |  |  | 9491 | 0.335 | 0.1761 | No |
| 106 | 30556 | tcf7l1b |  |  | 9704 | 0.321 | 0.1706 | No |
| 107 | 565155 | hsp90aa1.2 |  |  | 9737 | 0.319 | 0.1708 | No |
| 108 | 373103 | pak1 |  |  | 9754 | 0.318 | 0.1715 | No |
| 109 | 393664 | tuba8l2 |  |  | 9767 | 0.318 | 0.1723 | No |
| 110 | 100331324 | LOC100331324 |  |  | 9791 | 0.316 | 0.1727 | No |
| 111 | 336933 | rhogb |  |  | 9858 | 0.312 | 0.1718 | No |
| 112 | 373083\_1 | dynll2a |  |  | 9884 | 0.311 | 0.1722 | No |
| 113 | 562720 | mapk8a |  |  | 9888 | 0.310 | 0.1733 | No |
| 114 | 327377 | tuba4l |  |  | 9990 | 0.304 | 0.1712 | No |
| 115 | 101882851 | wu:fj66e11 |  |  | 10121 | 0.295 | 0.1682 | No |
| 116 | 402938 | txn2 |  |  | 10227 | 0.289 | 0.1659 | No |
| 117 | 566849 | cyth4a |  |  | 10239 | 0.289 | 0.1667 | No |
| 118 | 100330810 | nod1 |  |  | 10269 | 0.287 | 0.1668 | No |
| 119 | 65237\_1 | mapk14a |  |  | 10301 | 0.285 | 0.1669 | No |
| 120 | 394034 | waslb |  |  | 10311 | 0.284 | 0.1677 | No |
| 121 | 30523 | tcf7l1a |  |  | 10339 | 0.282 | 0.1679 | No |
| 122 | 557526 | traf2b |  |  | 10340 | 0.282 | 0.1690 | No |
| 123 | 641421 | tubb2b |  |  | 10342 | 0.282 | 0.1700 | No |
| 124 | 568951 | map2k4b |  |  | 10345 | 0.282 | 0.1710 | No |
| 125 | 406276 | s100a10b |  |  | 10413 | 0.278 | 0.1699 | No |
| 126 | 393141 | mycb |  |  | 10475 | 0.274 | 0.1690 | No |
| 127 | 336613 | cyfip1 |  |  | 10479 | 0.274 | 0.1700 | No |
| 128 | 563181 | map2k2a |  |  | 10792 | 0.256 | 0.1610 | No |
| 129 | 393550 | arpc5la |  |  | 10926 | 0.248 | 0.1577 | No |
| 130 | 406811\_2 | zgc:55461 |  |  | 11028 | 0.242 | 0.1554 | No |
| 131 | 100048921 | flnca |  |  | 11106 | 0.239 | 0.1539 | No |
| 132 | 403052 | map2k4a |  |  | 11173 | 0.234 | 0.1527 | No |
| 133 | 556424 | rhoga |  |  | 11444 | 0.220 | 0.1449 | No |
| 134 | 393139 | actr10 |  |  | 11498 | 0.217 | 0.1440 | No |
| 135 | 497649 | tcf7 |  |  | 11529 | 0.215 | 0.1439 | No |
| 136 | 100537448 | cyth3b |  |  | 11563 | 0.213 | 0.1437 | No |
| 137 | 114428 | arf1 |  |  | 11576 | 0.212 | 0.1441 | No |
| 138 | 405785 | tnfa |  |  | 11720 | 0.205 | 0.1403 | No |
| 139 | 556873 | si:ch211-175f11.5 |  |  | 11753 | 0.203 | 0.1401 | No |
| 140 | 100537244 | myl2a |  |  | 11772 | 0.202 | 0.1402 | No |
| 141 | 386701 | tubb5 |  |  | 11807 | 0.201 | 0.1399 | No |
| 142 | 556371 | rhoj |  |  | 11953 | 0.193 | 0.1360 | No |
| 143 | 556104 | arpc5lb |  |  | 11981 | 0.192 | 0.1359 | No |
| 144 | 767729 | dync1i2a |  |  | 12259 | 0.176 | 0.1278 | No |
| 145 | 317747 | rock2a |  |  | 12313 | 0.173 | 0.1267 | No |
| 146 | 431725 | zgc:91909 |  |  | 12321 | 0.172 | 0.1272 | No |
| 147 | 30510\_1 | tcf7l2 |  |  | 12361 | 0.171 | 0.1266 | No |
| 148 | 30707 | cse1l |  |  | 12382 | 0.170 | 0.1266 | No |
| 149 | 403139 | tlr5b |  |  | 12417 | 0.168 | 0.1261 | No |
| 150 | 494576\_1 | si:dkey-13a21.4 |  |  | 12522 | 0.163 | 0.1234 | No |
| 151 | 337737 | rab5aa |  |  | 12603 | 0.159 | 0.1215 | No |
| 152 | 368640 | kpna3 |  |  | 12628 | 0.158 | 0.1213 | No |
| 153 | 557302 | casp8l2 |  |  | 12635 | 0.157 | 0.1217 | No |
| 154 | 569226 | irak1 |  |  | 12707 | 0.154 | 0.1201 | No |
| 155 | 560569 | zgc:162964 |  |  | 12753 | 0.152 | 0.1192 | No |
| 156 | 368424 | snx9a |  |  | 12867 | 0.147 | 0.1162 | No |
| 157 | 559010 | exoc5 |  |  | 13041 | 0.138 | 0.1112 | No |
| 158 | 493612 | zgc:101810 |  |  | 13049 | 0.137 | 0.1115 | No |
| 159 | 559276 | ahnak |  |  | 13065 | 0.137 | 0.1115 | No |
| 160 | 100049706 | casp8l1 |  |  | 13088 | 0.136 | 0.1113 | No |
| 161 | 562750 | wasf3a |  |  | 13094 | 0.135 | 0.1117 | No |
| 162 | 336681 | tubb4b |  |  | 13139 | 0.133 | 0.1108 | No |
| 163 | 336063 | arf6a |  |  | 13195 | 0.130 | 0.1095 | No |
| 164 | 100329883 | fyco1b |  |  | 13392 | 0.121 | 0.1038 | No |
| 165 | 100001421 | snx33 |  |  | 13441 | 0.119 | 0.1027 | No |
| 166 | 394235 | pfn2 |  |  | 13511 | 0.115 | 0.1009 | No |
| 167 | 494163 | tab2 |  |  | 13529 | 0.114 | 0.1008 | No |
| 168 | 393902\_1 | rab7 |  |  | 13530 | 0.114 | 0.1013 | No |
| 169 | 394243 | arpc1a |  |  | 13546 | 0.113 | 0.1012 | No |
| 170 | 445305\_2 | arpc4l |  |  | 13646 | 0.109 | 0.0985 | No |
| 171 | 317743 | gapdh |  |  | 13695 | 0.107 | 0.0973 | No |
| 172 | 30681 | mapk12a |  |  | 13899 | 0.097 | 0.0913 | No |
| 173 | 569698 | mapk10 |  |  | 13907 | 0.096 | 0.0914 | No |
| 174 | 335474 | arpc1b |  |  | 13993 | 0.093 | 0.0891 | No |
| 175 | 403132\_1 | tlr4bb |  |  | 14024 | 0.091 | 0.0884 | No |
| 176 | 559866 | exoc4 |  |  | 14147 | 0.085 | 0.0849 | No |
| 177 | 100001191\_1 | si:ch211-147k9.8 |  |  | 14223 | 0.081 | 0.0828 | No |
| 178 | 573266\_1 | dync1li2 |  |  | 14230 | 0.081 | 0.0829 | No |
| 179 | 100149794 | akt3b |  |  | 14249 | 0.080 | 0.0827 | No |
| 180 | 405770 | il1b |  |  | 14290 | 0.079 | 0.0817 | No |
| 181 | 565852 | vps11 |  |  | 14325 | 0.077 | 0.0809 | No |
| 182 | 566639 | si:ch211-125o16.4 |  |  | 14366 | 0.076 | 0.0799 | No |
| 183 | 393716 | skp1 |  |  | 14456 | 0.071 | 0.0774 | No |
| 184 | 30701 | lef1 |  |  | 14523 | 0.068 | 0.0755 | No |
| 185 | 565756 | nfkb1 |  |  | 14558 | 0.067 | 0.0747 | No |
| 186 | 58081 | baxa |  |  | 14773 | 0.057 | 0.0681 | No |
| 187 | 266756 | mapk14b |  |  | 14849 | 0.053 | 0.0659 | No |
| 188 | 561894 | nckap1 |  |  | 14897 | 0.051 | 0.0646 | No |
| 189 | 562445 | arl8bb |  |  | 15007 | 0.046 | 0.0613 | No |
| 190 | 563632 | wasf3b |  |  | 15129 | 0.040 | 0.0576 | No |
| 191 | 58022 | casp8 |  |  | 15145 | 0.039 | 0.0573 | No |
| 192 | 406297 | dynll1 |  |  | 15150 | 0.039 | 0.0573 | No |
| 193 | 563215 | vps41 |  |  | 15244 | 0.035 | 0.0545 | No |
| 194 | 563365 | podxl |  |  | 15463 | 0.024 | 0.0477 | No |
| 195 | 100005026 | rilp |  |  | 15506 | 0.022 | 0.0464 | No |
| 196 | 415204 | arpc3 |  |  | 15534 | 0.020 | 0.0456 | No |
| 197 | 554861 | actr2b |  |  | 15691 | 0.012 | 0.0407 | No |
| 198 | 445164 | tuba8l3 |  |  | 15740 | 0.009 | 0.0392 | No |
| 199 | 406525 | dnm2b |  |  | 15794 | 0.007 | 0.0376 | No |
| 200 | 101884148\_1 | LOC101884148 |  |  | 15965 | -0.000 | 0.0322 | No |
| 201 | 799274 | kif5ab |  |  | 16053 | -0.003 | 0.0294 | No |
| 202 | 100885851 | il6 |  |  | 16069 | -0.004 | 0.0290 | No |
| 203 | 553413 | rhoh |  |  | 16095 | -0.005 | 0.0282 | No |
| 204 | 791141 | zgc:158659 |  |  | 16140 | -0.006 | 0.0268 | No |
| 205 | 503946 | fhod1 |  |  | 16236 | -0.009 | 0.0238 | No |
| 206 | 559334 | dnm2a |  |  | 16269 | -0.010 | 0.0229 | No |
| 207 | 334108 | kif5bb |  |  | 16350 | -0.012 | 0.0204 | No |
| 208 | 394163 | dynlrb1 |  |  | 16413 | -0.015 | 0.0185 | No |
| 209 | 100005446 | LOC100005446 |  |  | 16477 | -0.017 | 0.0165 | No |
| 210 | 321070 | rock2b |  |  | 16494 | -0.017 | 0.0161 | No |
| 211 | 406364 | elmo1 |  |  | 16544 | -0.019 | 0.0146 | No |
| 212 | 403125\_1 | tlr2 |  |  | 16599 | -0.020 | 0.0129 | No |
| 213 | 570772 | bcl2a |  |  | 16638 | -0.021 | 0.0118 | No |
| 214 | 100332402 | dync2h1 |  |  | 16726 | -0.023 | 0.0091 | No |
| 215 | 321383 | pfn2l |  |  | 16782 | -0.026 | 0.0075 | No |
| 216 | 751756 | rab9a |  |  | 17073 | -0.035 | -0.0016 | No |
| 217 | 767738 | abi1b |  |  | 17087 | -0.036 | -0.0019 | No |
| 218 | 30591 | hsp90aa1.1 |  |  | 17250 | -0.043 | -0.0068 | No |
| 219 | 30510 | tcf7l2 |  |  | 17360 | -0.048 | -0.0101 | No |
| 220 | 564403 | si:ch211-153j24.3 |  |  | 17386 | -0.049 | -0.0107 | No |
| 221 | 100009637 | wasla |  |  | 17655 | -0.061 | -0.0190 | No |
| 222 | 445472 | myo6b |  |  | 17671 | -0.061 | -0.0193 | No |
| 223 | 100005846 | si:dkey-56f14.7 |  |  | 17708 | -0.063 | -0.0202 | No |
| 224 | 324004 | ctnnb2 |  |  | 17720 | -0.064 | -0.0203 | No |
| 225 | 100149273 | mapk9 |  |  | 17865 | -0.070 | -0.0246 | No |
| 226 | 394174 | pik3cd |  |  | 17974 | -0.074 | -0.0277 | No |
| 227 | 566547 | tab3 |  |  | 17994 | -0.075 | -0.0281 | No |
| 228 | 406411 | rhoaa |  |  | 18330 | -0.091 | -0.0384 | No |
| 229 | 100534866 | flnb |  |  | 18363 | -0.092 | -0.0390 | No |
| 230 | 492355 | ralab |  |  | 18410 | -0.094 | -0.0401 | No |
| 231 | 565649 | fyco1a |  |  | 18448 | -0.096 | -0.0409 | No |
| 232 | 550458 | tubb6 |  |  | 18472 | -0.097 | -0.0413 | No |
| 233 | 324681 | rhogc |  |  | 18482 | -0.098 | -0.0412 | No |
| 234 | 101883172 | LOC101883172 |  |  | 18491 | -0.098 | -0.0411 | No |
| 235 | 450006 | myl9a |  |  | 18511 | -0.099 | -0.0413 | No |
| 236 | 767645 | zgc:153993 |  |  | 18516 | -0.099 | -0.0411 | No |
| 237 | 30265 | ctnnb1 |  |  | 18549 | -0.101 | -0.0417 | No |
| 238 | 399477 | arf2a |  |  | 18665 | -0.107 | -0.0450 | No |
| 239 | 415185 | mapk11 |  |  | 18679 | -0.107 | -0.0450 | No |
| 240 | 567938 | pik3c2b |  |  | 18707 | -0.109 | -0.0454 | No |
| 241 | 110440104 | LOC110440104 |  |  | 18765 | -0.112 | -0.0468 | No |
| 242 | 415099 | rela |  |  | 18848 | -0.116 | -0.0490 | No |
| 243 | 327204 | rac1a |  |  | 19053 | -0.124 | -0.0550 | No |
| 244 | 406681 | actr1 |  |  | 19140 | -0.128 | -0.0573 | No |
| 245 | 569358 | si:dkey-283b15.4 |  |  | 19157 | -0.129 | -0.0573 | No |
| 246 | 326719 | myl12.1 |  |  | 19292 | -0.135 | -0.0610 | No |
| 247 | 560549 | akt3a |  |  | 19324 | -0.136 | -0.0615 | No |
| 248 | 335499 | klc1b |  |  | 19335 | -0.137 | -0.0613 | No |
| 249 | 570288 | flncb |  |  | 19398 | -0.139 | -0.0627 | No |
| 250 | 393132 | irak4 |  |  | 19441 | -0.141 | -0.0636 | No |
| 251 | 399480 | mapk3 |  |  | 19514 | -0.144 | -0.0653 | No |
| 252 | 406728 | map2k1 |  |  | 19540 | -0.145 | -0.0655 | No |
| 253 | 393945 | rab5ab |  |  | 19731 | -0.154 | -0.0710 | No |
| 254 | 30573 | hsp90ab1 |  |  | 19796 | -0.157 | -0.0724 | No |
| 255 | 58130\_1 | tradd |  |  | 19801 | -0.157 | -0.0720 | No |
| 256 | 557109 | raf1b |  |  | 20203 | -0.175 | -0.0841 | No |
| 257 | 406471 | tnfrsf1a |  |  | 20265 | -0.178 | -0.0853 | No |
| 258 | 445473 | myo6a |  |  | 20344 | -0.181 | -0.0871 | No |
| 259 | 327373 | kpna4 |  |  | 20389 | -0.182 | -0.0878 | No |
| 260 | 572542 | kpna1 |  |  | 20404 | -0.183 | -0.0876 | No |
| 261 | 571950 | si:ch211-14c7.2 |  |  | 20468 | -0.186 | -0.0889 | No |
| 262 | 548603 | snx18b |  |  | 20477 | -0.186 | -0.0884 | No |
| 263 | 336839 | cdc42 |  |  | 20515 | -0.189 | -0.0889 | No |
| 264 | 100002872\_1 | cyfip2 |  |  | 20559 | -0.191 | -0.0895 | No |
| 265 | 553542 | zgc:109889 |  |  | 20617 | -0.194 | -0.0906 | No |
| 266 | 554127 | tubb2 |  |  | 20659 | -0.196 | -0.0912 | No |
| 267 | 494576 | si:dkey-13a21.4 |  |  | 20660 | -0.197 | -0.0904 | No |
| 268 | 337084 | myl12.2 |  |  | 20670 | -0.197 | -0.0900 | No |
| 269 | 373093 | hdr |  |  | 20727 | -0.200 | -0.0910 | No |
| 270 | 336550 | rps3 |  |  | 20858 | -0.207 | -0.0944 | No |
| 271 | 100009637\_1 | wasla |  |  | 21002 | -0.216 | -0.0981 | No |
| 272 | 415187 | brk1 |  |  | 21013 | -0.216 | -0.0976 | No |
| 273 | 335110 | dync1h1 |  |  | 21161 | -0.224 | -0.1014 | No |
| 274 | 327026 | arf2b |  |  | 21311 | -0.231 | -0.1053 | No |
| 275 | 335798 | zgc:65894 |  |  | 21348 | -0.234 | -0.1055 | No |
| 276 | 431767 | dctn3 |  |  | 21436 | -0.238 | -0.1074 | No |
| 277 | 436770 | dctn5 |  |  | 21459 | -0.238 | -0.1072 | No |
| 278 | 325586\_1 | cyth1b |  |  | 21476 | -0.240 | -0.1068 | No |
| 279 | 393653 | rhogd |  |  | 21640 | -0.248 | -0.1111 | No |
| 280 | 399482 | arpc5b |  |  | 21672 | -0.250 | -0.1111 | No |
| 281 | 436866 | tnfsf10 |  |  | 21725 | -0.253 | -0.1118 | No |
| 282 | 560840 | pkn1a |  |  | 21736 | -0.253 | -0.1112 | No |
| 283 | 405821\_1 | rab5b |  |  | 21939 | -0.264 | -0.1166 | No |
| 284 | 541530 | ikbkg |  |  | 21943 | -0.264 | -0.1157 | No |
| 285 | 556640 | si:ch211-69b7.6 |  |  | 21992 | -0.267 | -0.1162 | No |
| 286 | 562365 | klc2 |  |  | 22099 | -0.273 | -0.1186 | No |
| 287 | 406303 | tuba2 |  |  | 22113 | -0.274 | -0.1179 | No |
| 288 | 554561 | traf6 |  |  | 22116 | -0.274 | -0.1170 | No |
| 289 | 556909 | fbxo22 |  |  | 22117 | -0.274 | -0.1159 | No |
| 290 | 100038776 | actr3b |  |  | 22285 | -0.283 | -0.1202 | No |
| 291 | 567078 | vps39 |  |  | 22297 | -0.284 | -0.1195 | No |
| 292 | 569394 | klc3 |  |  | 22448 | -0.293 | -0.1231 | No |
| 293 | 403084 | tab1 |  |  | 22472 | -0.294 | -0.1227 | No |
| 294 | 406250 | actr3 |  |  | 22666 | -0.305 | -0.1277 | No |
| 295 | 325557 | anxa2a |  |  | 22803 | -0.314 | -0.1309 | No |
| 296 | 563459 | kif5ba |  |  | 22998 | -0.327 | -0.1358 | No |
| 297 | 567460 | ripk1l |  |  | 23090 | -0.333 | -0.1374 | No |
| 298 | 337424 | arl8ba |  |  | 23120 | -0.335 | -0.1371 | No |
| 299 | 406659 | rhoac |  |  | 23154 | -0.338 | -0.1369 | No |
| 300 | 406493 | myl9b |  |  | 23170 | -0.339 | -0.1361 | No |
| 301 | 561525 | dync1i1 |  |  | 23257 | -0.345 | -0.1375 | No |
| 302 | 100006250 | LOC100006250 |  |  | 23302 | -0.347 | -0.1376 | No |
| 303 | 564823 | plekhm2 |  |  | 23316 | -0.348 | -0.1367 | No |
| 304 | novel.934 | - |  |  | 23418 | -0.356 | -0.1385 | No |
| 305 | 360144\_1 | mapk1 |  |  | 23446 | -0.357 | -0.1381 | No |
| 306 | 550479 | dctn4 |  |  | 23495 | -0.360 | -0.1382 | No |
| 307 | 100002263 | pik3cb |  |  | 23508 | -0.361 | -0.1373 | No |
| 308 | 795591 | si:ch211-114n24.6 |  |  | 23557 | -0.364 | -0.1374 | No |
| 309 | 566086 | kif5aa |  |  | 23616 | -0.368 | -0.1379 | No |
| 310 | 795051 | LOC795051 |  |  | 23678 | -0.372 | -0.1384 | No |
| 311 | 65237 | mapk14a |  |  | 23905 | -0.386 | -0.1441 | No |
| 312 | 100004555 | ripk3 |  |  | 23949 | -0.389 | -0.1440 | No |
| 313 | 100003026 | dctn1b |  |  | 24106 | -0.400 | -0.1475 | No |
| 314 | 100003206 | arf6b |  |  | 24135 | -0.402 | -0.1468 | No |
| 315 | 548346 | pik3c3 |  |  | 24150 | -0.403 | -0.1458 | No |
| 316 | 564048\_1 | plekhm1 |  |  | 24210 | -0.407 | -0.1461 | No |
| 317 | 101884148 | LOC101884148 |  |  | 24339 | -0.415 | -0.1486 | No |
| 318 | 394141 | dctn2 |  |  | 24688 | -0.429 | -0.1581 | No |
| 319 | 100002225 | map2k2b |  |  | 24736 | -0.433 | -0.1579 | No |
| 320 | 566639\_1 | si:ch211-125o16.4 |  |  | 24755 | -0.435 | -0.1569 | No |
| 321 | 100006041 | rhoab |  |  | 24773 | -0.436 | -0.1557 | No |
| 322 | 550569 | myl10 |  |  | 24980 | -0.444 | -0.1606 | No |
| 323 | 393287 | chuk |  |  | 25038 | -0.449 | -0.1607 | No |
| 324 | 327068 | arpc2 |  |  | 25071 | -0.452 | -0.1601 | No |
| 325 | 406723 | exoc7 |  |  | 25188 | -0.459 | -0.1620 | No |
| 326 | novel.818 | - |  |  | 25208 | -0.460 | -0.1609 | No |
| 327 | 415158 | cycsb |  |  | 25213 | -0.461 | -0.1593 | No |
| 328 | 556640\_1 | si:ch211-69b7.6 |  |  | 25277 | -0.465 | -0.1595 | No |
| 329 | 556951 | zgc:172352 |  |  | 25330 | -0.469 | -0.1594 | No |
| 330 | 101886083 | LOC101886083 |  |  | 25338 | -0.469 | -0.1579 | No |
| 331 | 100006857 | elmo2 |  |  | 25345 | -0.470 | -0.1563 | No |
| 332 | 567222 | arl8a |  |  | 25507 | -0.485 | -0.1596 | No |
| 333 | 57935 | actb2 |  |  | 25530 | -0.487 | -0.1584 | No |
| 334 | 557810 | mapk12b |  |  | 25647 | -0.498 | -0.1602 | No |
| 335 | 57933 | caspa |  |  | 25658 | -0.499 | -0.1587 | No |
| 336 | 553413\_1 | rhoh |  |  | 25660 | -0.499 | -0.1568 | No |
| 337 | 564261\_1 | si:rp71-17i16.5 |  |  | 25681 | -0.501 | -0.1556 | No |
| 338 | 393928 | klc1a |  |  | 25735 | -0.506 | -0.1554 | No |
| 339 | 30716 | raf1a |  |  | 25882 | -0.518 | -0.1580 | No |
| 340 | 403138 | tlr5a |  |  | 26038 | -0.532 | -0.1610 | No |
| 341 | 562317 | fadd |  |  | 26068 | -0.535 | -0.1599 | No |
| 342 | 101886383 | LOC101886383 |  |  | 26396 | -0.567 | -0.1681 | No |
| 343 | 556103 | dync1li1 |  |  | 26421 | -0.569 | -0.1667 | No |
| 344 | 550286 | hrasa |  |  | 26472 | -0.575 | -0.1662 | No |
| 345 | 448942\_1 | vps33a |  |  | 26570 | -0.585 | -0.1670 | No |
| 346 | 373083 | dynll2a |  |  | 26592 | -0.587 | -0.1655 | No |
| 347 | 415190 | zgc:86896 |  |  | 26776 | -0.607 | -0.1690 | No |
| 348 | 100005887 | vps18 |  |  | 26796 | -0.609 | -0.1673 | No |
| 349 | 100002190 | cyth3a |  |  | 26874 | -0.617 | -0.1675 | No |
| 350 | 447930 | mylpfb |  |  | 27171 | -0.644 | -0.1744 | No |
| 351 | 553656 | hrasb |  |  | 27554 | -0.680 | -0.1840 | No |
| 352 | 321938 | cyth1a |  |  | 27695 | -0.700 | -0.1858 | No |
| 353 | 103911411\_1 | LOC103911411 |  |  | 27699 | -0.701 | -0.1833 | No |
| 354 | 110438182 | LOC110438182 |  |  | 28145 | -0.763 | -0.1945 | No |
| 355 | 562838 | rac1b |  |  | 28146 | -0.763 | -0.1917 | No |
| 356 | 556226 | cdc42l2 |  |  | 28186 | -0.770 | -0.1900 | No |
| 357 | 799355 | pfn1 |  |  | 28251 | -0.778 | -0.1891 | No |
| 358 | 100141338 | cyth4b |  |  | 28259 | -0.779 | -0.1864 | No |
| 359 | 558552 | tubb1 |  |  | 28296 | -0.785 | -0.1846 | No |
| 360 | 403131\_1 | tlr4ba |  |  | 28357 | -0.796 | -0.1835 | No |
| 361 | 101910198 | akt1 |  |  | 28441 | -0.810 | -0.1831 | No |
| 362 | 100001191 | si:ch211-147k9.8 |  |  | 28494 | -0.818 | -0.1816 | No |
| 363 | 565740 | dynlt1 |  |  | 28568 | -0.834 | -0.1808 | No |
| 364 | 393993 | ralaa |  |  | 28607 | -0.840 | -0.1789 | No |
| 365 | 100006208 | LOC100006208 |  |  | 28738 | -0.866 | -0.1797 | No |
| 366 | 767746 | zgc:153426 |  |  | 28740 | -0.866 | -0.1765 | No |
| 367 | 799806\_2 | anxa2b |  |  | 28928 | -0.905 | -0.1790 | No |
| 368 | 100006166 | si:ch211-1f22.1 |  |  | 28986 | -0.917 | -0.1774 | No |
| 369 | 553634 | casp7 |  |  | 29151 | -0.959 | -0.1790 | No |
| 370 | 114456 | arpc4 |  |  | 29163 | -0.962 | -0.1757 | No |
| 371 | 561612 | dync1i2b |  |  | 29202 | -0.974 | -0.1732 | No |
| 372 | 100007048 | bcl2b |  |  | 29242 | -0.987 | -0.1708 | No |
| 373 | 558917 | snx9b |  |  | 29245 | -0.988 | -0.1671 | No |
| 374 | 325586 | cyth1b |  |  | 29278 | -0.995 | -0.1644 | No |
| 375 | 403146 | akt2l |  |  | 29342 | -1.012 | -0.1626 | No |
| 376 | 100003911 | cxcl19 |  |  | 29359 | -1.017 | -0.1592 | No |
| 377 | 573122 | tuba1c |  |  | 29368 | -1.020 | -0.1557 | No |
| 378 | 798445 | LOC798445 |  |  | 29435 | -1.044 | -0.1538 | No |
| 379 | 449549 | zgc:100918 |  |  | 29484 | -1.058 | -0.1514 | No |
| 380 | 561021\_1 | si:dkey-103e21.5 |  |  | 29577 | -1.086 | -0.1502 | No |
| 381 | 101884327 | LOC101884327 |  |  | 29590 | -1.090 | -0.1465 | No |
| 382 | 30592 | myl7 |  |  | 29608 | -1.098 | -0.1429 | No |
| 383 | 445305\_1 | arpc4l |  |  | 29885 | -1.197 | -0.1471 | No |
| 384 | 792699 | rac1l |  |  | 30004 | -1.247 | -0.1462 | No |
| 385 | 100002872 | cyfip2 |  |  | 30090 | -1.280 | -0.1441 | No |
| 386 | 100002946 | cxcl8a |  |  | 30532 | -1.528 | -0.1523 | No |
| 387 | 571356\_1 | pik3c2a |  |  | 30618 | -1.605 | -0.1490 | No |
| 388 | 100004870 | exoc2 |  |  | 30650 | -1.633 | -0.1438 | No |
| 389 | 562372 | snx18a |  |  | 30706 | -1.679 | -0.1392 | No |
| 390 | 101883269 | LOC101883269 |  |  | 30732 | -1.709 | -0.1336 | No |
| 391 | 563023 | flnb |  |  | 30744 | -1.716 | -0.1275 | No |
| 392 | 140621\_1 | casp3a |  |  | 30781 | -1.743 | -0.1221 | No |
| 393 | 798985 | vps16 |  |  | 30791 | -1.750 | -0.1158 | No |
| 394 | 406811\_1 | zgc:55461 |  |  | 30849 | -1.809 | -0.1108 | No |
| 395 | 57934 | actb1 |  |  | 31071 | -2.048 | -0.1101 | No |
| 396 | 436734\_1 | txn |  |  | 31085 | -2.064 | -0.1027 | No |
| 397 | 564295\_2 | traf2a |  |  | 31089 | -2.074 | -0.0950 | No |
| 398 | novel.848 | - |  |  | 31248 | -2.325 | -0.0912 | No |
| 399 | 562529\_2 | flna |  |  | 31348 | -2.521 | -0.0849 | No |
| 400 | 30429\_2 | mylpfa |  |  | 31431 | -2.730 | -0.0772 | No |
| 401 | 386590 | hsp90b1 |  |  | 31551 | -3.061 | -0.0695 | No |
| 402 | 140621 | casp3a |  |  | 31691 | -3.652 | -0.0601 | No |
| 403 | 403131 | tlr4ba |  |  | 31734 | -3.963 | -0.0465 | No |
| 404 | 436718\_1 | rhocb |  |  | 31770 | -4.295 | -0.0315 | No |
| 405 | 793158 | cdc42l |  |  | 31794 | -4.556 | -0.0151 | No |
| 406 | 373107\_1 | birc2 |  |  | 31825 | -4.799 | 0.0021 | No |
Table: GSEA details [plain text format]

  

Fig 2: SALMONELLA\_INFECTION(DRE05132)      
 Blue-Pink O' Gram in the Space of the Analyzed GeneSet

  

Fig 3: SALMONELLA\_INFECTION(DRE05132): Random ES distribution      
 Gene set null distribution of ES for **SALMONELLA\_INFECTION(DRE05132)**
